# A prospective, randomized, double-blind trial to compare body weight-adjusted and fixed doses of palonosetron for preventing postoperative nausea and vomiting in obese female patients

**DOI:** 10.1371/journal.pone.0227490

**Published:** 2020-01-14

**Authors:** Nathalia Gouveia de Araujo Ferreira, Ismar Lima Cavalcanti, Alexandra Rezende Assad, Louis Barrucand, Estêvão Luiz Carvalho Braga, Nubia Verçosa

**Affiliations:** 1 Department of Anesthesiology, National Cancer Institute (HCIII), Rio de Janeiro, Rio de Janeiro, Brazil; 2 Postgraduate Program Surgical Sciences, Federal University of Rio de Janeiro, Rio de Janeiro, Rio de Janeiro, Brazil; 3 Department of General and Specialized Surgery/Anesthesiology, Fluminense Federal University, Niterói, Rio de Janeiro, Brazil; 4 Faculty of Medicine, Federal University of Rio de Janeiro, Rio de Janeiro, Brazil; 5 Department of Surgery/Anesthesiology, Postgraduate Program Surgical Sciences, Federal University of Rio de Janeiro, Rio de Janeiro, Brazil; Vanderbilt University, UNITED STATES

## Abstract

**Background:**

Postoperative nausea and vomiting (PONV) is a common postsurgical complication. Palonosetron is effective for PONV prevention at the usual dose of 75 μg, but the ideal dose for obese patients has not yet been investigated. The aim of this study was to compare body weight-adjusted and fixed doses of palonosetron for preventing PONV in obese female patients.

**Materials and methods:**

We performed a prospective, randomized, double-blind trial involving 80 female patients, aged 18–80 years with an American Society of Anesthesiologists physical status of 2 and 3 and a body mass index (BMI) ≥ 30 kg m^-2^ who were scheduled to undergo elective breast surgery. Patients received an intravenous body weight-adjusted dose of palonosetron (1 μg kg ^-1^, GI = 40 patients) or a fixed dose of palonosetron (75 μg, GII = 40 patients). All patients received dexamethasone (4 mg). The incidence of PONV, complete response rate (CR), severity of nausea and need for rescue antiemetics and analgesics were assessed at: 0–1 h, 1–6 h, 6–24 h and 24–48 h postoperatively.

**Results:**

The mean (± SD) BMI was 35.0 (±5.2) kg m^-2^ for GI and 35.7 (±3.6) kg m^-2^ for GII. There was no significant difference between groups in PONV incidence, CR, severity of nausea, and need for rescue antiemetics or analgesics. The incidence of PONV for GI and GII was 15% and 27.5%, respectively, during the first 48 h (P = 0.17).

**Conclusions:**

A body weight-adjusted dose of palonosetron was as effective as 75 μg for preventing PONV for 48 h in obese female patients who underwent breast surgery. Hence, the fixed dose may be preferable to the body weight-adjusted dose.

## Introduction

Preventing postoperative nausea and vomiting (PONV) is routine during anesthesia/surgical procedures, since PONV is a frequent complication with an incidence of 30–50%, reaching up to 80% in high-risk patients [1,2]. PONV is associated with discomfort and morbidities such as dehydration, esophageal rupture, wound dehiscence, bleeding, pulmonary aspiration and prolonged hospital stays [3,4]. Serotonin antagonists are largely used for PONV prophylaxis and act by inhibiting the calcium influx caused by stimulation of the 5-hydroxytryptamine-type 3 receptor (5-HT_3_) [5–7]. Palonosetron is a second-generation 5-HT_3_ antagonist with a higher binding affinity and longer half-life of 40 h than other 5-HT_3_ antagonists [8,9]. Initially, palonosetron was used to prevent nausea and vomiting induced by chemotherapy [10], but it has also been proven effective for preventing PONV [7,11], usually at a dose of 75 μg. Although a few trials have evaluated body weight-adjusted doses [12,13], the ideal dose for obese patients has not yet been investigated.

Our hypothesis was that a body weight-adjusted dose of palonosetron (1 μg kg ^-1^) would be more effective than a fixed dose (75 μg) for preventing PONV in obese female patients. The aim of this study was to compare the incidence of PONV, complete response rate (CR), severity of nausea and need for rescue antiemetics or analgesics associated with two different doses of palonosetron.

## Materials and methods

We performed a prospective, randomized, double-blind, single center clinical trial with obese female patients undergoing elective breast surgery in a secondary hospital dedicated to cancer treatment between November 2016 and February 2018. This study was approved by the Research Ethics Committee of the National Institute of Cancer, Rio de Janeiro, Brazil (CAAE: 55695816.7.0000.5274). The trial was registered prior to patient enrolment at ClinicalTrials.gov (NCT02941913; principal investigator: Nathalia Gouveia de Araujo Ferreira; date of registration: October 19, 2016). Written informed consent was obtained from all patients prior to surgery. This manuscript adheres to the applicable CONSORT guidelines.

The primary outcome was PONV incidence during the first 48 h postoperatively. The secondary outcomes were the CR (i.e., patients who neither vomited nor required antiemetic rescue medication for nausea), severity of nausea and the need for rescue antiemetics and analgesics as assessed during 4 periods: 0–1 h, 1–6 h, 6–24 h and 24–48 h postoperatively.

Nausea was defined as a subjective unpleasant sensation associated with the urge to vomit without expulsion of gastric contents, and vomiting was defined as the forceful expulsion of gastric contents through the mouth. We recorded nausea severity using a verbal scale as follows: no nausea, mild, moderate and severe.

All patients were assessed by the Apfel criteria [14] for their risk factors for PONV (female gender, prior history of motion sickness or PONV, non-smoking and the use of postoperative opioids).

Eighty patients were recruited according to the following inclusion criteria: female, age between 18–80 years old, American Society of Anesthesiologists (ASA) physical status of 2 and 3, body mass index (BMI) equal to or greater than 30 kg m^-2^ and elective breast surgery (segmental mastectomy, simple mastectomy and modified radical mastectomy). The exclusion criteria were the occurrence of nausea or vomiting episodes in the last 24 h prior to surgery, the use of corticosteroids, smoking, alcoholism, the use of psychoactive drugs or any other drug with antiemetic effects, hypersensitivity to other 5-HT_3_ antagonists, emergency surgeries and chemotherapy within 4 weeks.

Using computer-generated random numbers (GraphPad Prism Quickcalcs Software^®^, Inc., La Jolla, CA, USA), the patients were assigned with sealed opaque envelopes into one of the two treatment groups on the morning of surgery. Forty patients received a body weight-adjusted dose of intravenous palonosetron at 1 μg kg ^-1^ (group GI), and another forty patients received a fixed dose of intravenous palonosetron at 75 μg (group GII). Each study drug was mixed with saline to a total volume of 10 ml in an unlabeled syringe by an anesthesiologist not involved in the study. All patients and anesthesiologists involved in the study were blinded to the group allocation to maintain double-blind conditions.

Premedication with midazolam (15 mg orally) was administered the morning of surgery. Routine monitoring consisted of electrocardiography, noninvasive blood pressure assessment, capnography, pulse oximetry, peripheral nerve stimulation (Cardiocap 5 Datex-Ohmeda, Helsinki, Finland) and bispectral index (BIS, A-1050 Monitor, Aspect Medical Systems, Newton, MA, USA). After placing an intravenous cannula (20 G or 18 G), palonosetron (ONICIT^®^—Pierre Fabre Medicament Production, Idron, France—glass vial contains 0.075 mg (free base) in 1.5 mL -concentration: 0.050 mg mL^-1^) was administered one minute before induction as an intravenous bolus. The patients were preoxygenated with oxygen at 6 L min^-1^ using a face mask for 5 minutes followed by intravenous administration of propofol 1.5 mg kg^-1^, fentanyl 3 μg kg^-1^, lidocaine 1.5 mg kg^-1^ and rocuronium 0.3 mg kg^-1^. A laryngeal mask was inserted, and pulmonary ventilation was maintained with a tidal volume of 6 mL kg^-1^ of ideal weight and a maximum peak pressure of 25 cm H_2_O. Intravenous dexamethasone (4 mg) was administered. Anesthesia was maintained with sevoflurane in 50% oxygen/air (2 L min^-1^) with the concentration adjusted to ensure an equal depth of anesthesia during surgery, as assessed by BIS maintained between 40–60. Remifentanil (0.05 to 0.2 μg kg ^-1^ min^-1^) was administered for supplemental intraoperative analgesia if needed and the dose was adjusted to maintain blood pressure and heart rate within 20% of baseline values. Additional doses of rocuronium were administered as needed to maintain moderate neuromuscular blockade (train of four (TOF), count of 2). For postoperative analgesia, all patients received the nonsteroidal anti-inflammatory drug (NSAID) tenoxicam 40 mg, metamizole (dipyrone) 50 mg kg^-1^ and the opioid tramadol 100 mg intravenously 40 minutes before the end of surgery. Residual neuromuscular blockade was reversed with sugammadex 2 mg kg ^-1^. Patients stayed in the Post-Anesthesia Care Unit (PACU) for at least 1 h before they were transferred to the hospital ward. The patients were discharged from the hospital 24 h postoperatively.

Metoclopramide 10 mg was given to both groups as an antiemetic rescue medication intravenously during the hospital stay and orally after hospital discharge for the first 48 h postoperatively.

For postoperative analgesic control, the institutional pain protocol was used, which established the administration of tramadol (100 mg, intravenous, every 8 hours) in cases of pain with a visual analogue scale score greater than 3.

Patients were assessed at 4 time periods: 0–1 h, 1–6 h, 6–24 h and 24–48 h postoperatively to record the incidence of PONV episodes, CR, severity of nausea and the need for rescue analgesics and antiemetics. After discharge, all patients were contacted by telephone over a period of 24–48 h.

### Statistical analysis

Shapiro-Wilk test for normality was applied to all variable samples of the demographic data. The calculated W value for all samples that were higher than the one recommended by the test and corresponding to P = 0.10 was accepted as normal. All variables of both groups did not reach normality. From the nonparametric Wilcoxon-Mann-Whitney test the Z-test was used due to the large sample sizes and the values obtained converted into P values from the normal distribution table for ½ P.

For categorical data, inter and intra group comparison was performed using the Fisher’s exact test for 2x2 contingency table. The value of P ≤ 0.05 was considered statistically significant.

The study was powered at 90% to detect a difference of PONV of 48% to 15% at alpha = 0.05 with 40 patients per group. This calculation is for the support of Table 2 (ASA variables and other diseases) and Table 3 (Incidence of PONV).

## Results

**Fig 1:**
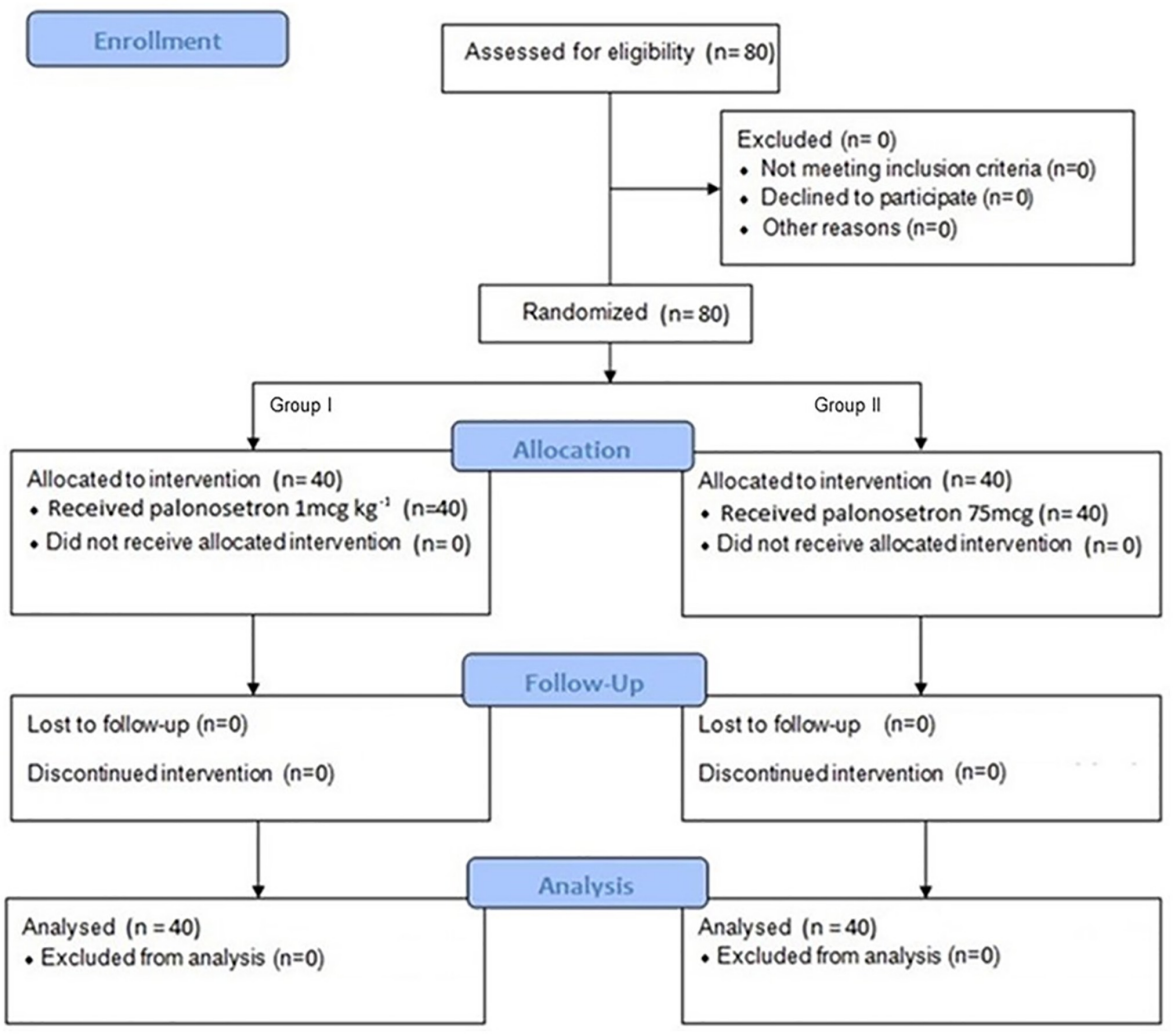
Flow chart of the study—CONSORT.

A total of 80 eligible female patients were recruited and randomized into two groups: 40 for the GI group (1 μg kg^-1^ of palonosetron) and 40 for the GII group (75 μg of palonosetron) “Fig 1”.

The demographic data of the enrolled patients are shown in “Table 1”. There was no significant difference regarding age, weight or BMI between the groups.

**Table 1:** Demographic data: Age, weight and body mass index.

Variable		N	Min	Percentage			Max	Mean	SD	Z-score	P (*)
				25%	Median	75%					(2 tailed)
Age (years)	GI	40	31.0	45.0	54.0	58.5	70.0	53.0	9.4	1.87	0.33
	GII	40	38.0	46.0	58.0	64.5	80.0	56.5	10.4		
Weight (kg)	GI	40	76.0	81.0	91.5	99.5	126.0	92.7	13.3	0.96	0.33
	GII	40	75.0	82.5	88.0	94.0	115.0	89.0	9.3		
BMI (kg m^-2^)	GI	40	30.4	32.1	35.0	39.6	53.0	36.3	5.2	0.33	0.74
	GII	40	30.3	34.1	35.7	38.0	47.8	36.0	3.6		

Data are expressed as the mean ± SD, minimum (min), maximum (max) or median. GI: palonosetron 1 μg kg^-1^; GII: palonosetron 75 μg; BMI: body mass index; P- statistical significance. Z-score from Shapiro test; (*) Mann-Whitney test.

The mean dose used in the GI group was 92.7 μg (± 13.3), and in the GII group, a fixed dose of 75 μg was used (P = 0.16).

There was no significant difference in the ASA status or associated diseases, as shown in “Table 2”.

**Table 2:** ASA and associated diseases.

Variable	GI (%) (n = 40)	GII (%) (n = 40)	P (*) (2-tailed)
ASA 2	11 (27.5%)	9 (22.5%)	0.80
ASA 3	29 (72.5%)	31 (77.5%)	0.65
Hypertension	25 (62.5%)	29 (72.5%)	0.43
Diabetes mellitus	13 (32.5%)	13 (32.5%)	1.00
Others (+)	10 (25.0%)	5 (12.5%)	0.58

Data are the numbers (%). GI: palonosetron 1 μg kg^-1^; GII: palonosetron 75 μg; ASA: American Society of Anesthesiologists physical status; (+) hypo and hyperthyroidism; gastritis; coronary arterial heart disease; asthma and psoriasis; (*) MedCal test using GI and GII and corresponding sample size proportion.

There was no statistically significant difference between the two groups regarding the surgery time, fluid requirements, fentanyl dose, remifentanil dose or fasting time.

The incidence of PONV at 0–1 h, 1–6 h, 6–24 h and 24–48 h after surgery did not differ significantly between the groups “Table 3”.

**Table 3:** Incidence of nausea, vomiting and PONV in the groups.

Variable	GI (%) (n = 40)	GII (%) (n = 40)	P (2 tailed)
Nausea			
0 to 1 h	4 (10.0)	3 (7.5)	0.69
1 to 6 h	1 (2.5)	1 (2.5)	1.00
6 to 24 h	0	2 (5.0)	1.00
24 to 48 h	2 (5.0)	5 (12.5)	0.43
Overall 0 to 48 h	6 (15.0)	11 (27.5)	0.17
Vomiting			
0 to 1 h	0	1 (2.5)	0.3
1 to 6 h	0	0	0
6 to 24 h	0	0	0
24 to 48 h	0	0	0
Overall 0 to 48 h	0	1 (2.5)	0.3
PONV			
0 to 1 h	4 (10.0)	3 (7.5)	0.69
1 to 6 h	1 (2.5)	1 (2.5)	1.00
6 to 24 h	0	2 (5.0)	1.00
24 to 48 h	2 (5.0)	5 (12.5)	0.43
Overall 0 to 48 h	6 (15.0)	11 (27.5)	0.17

Data are the numbers (%). GI: palonosetron 1 μg kg^-1^; GII: palonosetron 75 μg; PONV: postoperative nausea and vomiting; % percentage. The statistical calculator MedCal was used using GI and GII and corresponding sample size proportion.

One patient from the GI group had recurrent episodes of nausea, with the first episode occurring 1–6 h postoperatively and the other between 24–48 h. One patient from the GII group developed vomiting, which was preceded by nausea within 0 to 1 h postoperatively. All patients with nausea reported mild symptoms.

The total incidence of PONV in our study, including both groups, in the first 48 h was 21.25%. The incidence of PONV between 0 and 48 h was 15% for the GI group and 27.5% for the GII group (P = 0.17). The incidence of PONV between 24 h and 48 h after surgery was lower in the GI group than in the GII group (5% vs 12.5%), but the difference was not statistically significant (P = 0.43) “Table 3”.

The CR was similar between the groups. In the 0–48 h postoperative interval, the CR was 85% (34 patients) for the GI group and 72.5% (29 patients) for the GII group (P = 0.17). From 0–24 h, the CR was 87.5% (35 patients) for the GI group and 85% (34 patients) for the GII group (P = 0.74), while from 24–48 h, the CR was 95% (38 patients) and 87.5% (35 patients) in the GI and GII groups (P = 0.23), respectively.

All patients who experienced PONV received rescue antiemetics, so the use of antiemetics did not differ significantly among the groups.

The need for the rescue analgesic opioid was similar between the groups: a single dose of tramadol (100 mg intravenous) was used in 3 patients in the GI group (7.5%) and 4 patients in the GII group (10%). All patients required rescue doses during the first 6 h postoperatively.

Regarding the risk factors for PONV, the Apfel score was similar between the groups (P = 0.26). All enrolled patients had at least 2 risk factors because they were nonsmoking females. Two and 6 patients from the GI and GII groups, respectively, had Apfel scores of 3 because they had a history of PONV or required postoperative opioids (P = 0.33). Only one patient from the GI group had an Apfel score of 4 since she had a history of PONV and required postoperative opioids. Fifty-five percent of the patients with Apfel scores of 3–4 developed PONV postoperatively.

In this study, palonosetron was well tolerated. No adverse events (such as pruritus, dizziness, headache or constipation) caused by the studied drug were recorded.

## Discussion

This prospective, randomized, controlled trial with 80 obese female patients demonstrated that body weight-adjusted (1 μg kg ^-1^) and fixed doses of palonosetron (75 μg) were equally effective for the prevention of PONV during the first 48 h postoperatively.

As no research was found in the literature using Palonosetron in obese patients this work seems to be original.

In the present study, only non-smoking female patients were recruited because this population has a high risk of PONV [1,15]. The impact of body weight on determining the dose of palonosetron has not yet been investigated; therefore, only obese patients were enrolled. A body weight-adjusted dose of 1 μg kg^-1^ was proposed based on other dosing schedule studies [12,13]. The latter study [13], in which body weight-adjusted doses of palonosetron of 0.5, 1.0, and 1.5 μg kg^-1^ were used, enrolled a population with the average weight of 60 kg, resulting in mean doses of 30, 60 and 90 μg, respectively. This study showed a dose-dependent prophylactic effect. Since the present study involved an obese population with an average weight of 90 kg, the dose of 1 μg kg^-1^ would have led to an average dose of 90 μg. Based on this measurement, we decided to use the dose of 1 μg kg^-1^. Greater doses in obese patients could lead to greater risks of side effects and greater costs. Therefore, the study protocol established 1 μg kg^-1^ for the body weight-adjusted dose.

The total incidence of PONV in our study was 21.25%, which is consistent with the results of other studies in the literature [3,5,16–18]. During the first postoperative 24 h, the incidence of PONV was also comparable to other study results [17,19]. After 24 h, the incidence of PONV was lower in the GI group (5%) than in the GII group (12.5%), although the difference was not statistically significant (P = 0.43). This finding suggests that the weight-adjusted dose has an antiemetic effect that lasts longer than the fixed dose.

Previous studies [2, 20,21] have reported that 5-HT_3_ receptor antagonists may be better antiemetics than antinausea agents, which can explain the much lower incidence of vomiting (1.25%) than the incidence of nausea (21.25%) in this study.

The CR found in the different periods of our study are in accord with those of another study [16] that reported a CR of 85.7% from 0–24 h postoperatively and 100% from 24–48 h postoperatively in a palonosetron-dexamethasone group [16]. Similar results were reported in a different study [22] that showed a CR of 90% over 48 h postoperatively.

Only 8.75% of the patients in our study received postoperative opioid analgesics, which is an important risk factor for PONV. Several studies [2,23,24] that found a higher incidence of PONV reported that patients used postoperative opioids more frequently. In our study, the type of surgery was associated with a low pain score; thus, the low postoperative opioid consumption may have led to a lower incidence of PONV. PONV is closely related to effective pain control, which was the reason we chose to use an identical multimodal pain control protocol in both groups [18].

Patients enrolled in this study were considered to be at high risk for PONV because they all had at least two risk factors according the Apfel criteria (females and nonsmokers); in addition, these patients had received volatile anesthetics [25]. With regard to high-risk patients, the literature [1,25] recommends combination antiemetic therapy; therefore, dexamethasone was used as a second prophylactic intervention for all patients in our study. The benefits of using dexamethasone with palonosetron have been demonstrated in some studies [16,26]. Considering our high-risk patients, a high incidence of PONV would be expected, as shown in other studies [2,7,13,23,27] with a similar population design that did not use multimodal antiemetic prophylactic therapy. Based on the low incidence of PONV, our study seems to suggest that PONV prevention is improved by the combination of dexamethasone with palonosetron.

Although younger age (< 50 years) has been found to be a risk factor for PONV in the literature [1], in our study, the measurement of the risk factors for PONV was made using the Apfel criteria, which does not include age as a risk factor. Additionally, our population consisted of patients with breast cancer, which is more frequent in patients greater than 50 years of age; thus, our study had a broader age range.

A previous study [27] of increasing doses of palonosetron showed that 75 μg was better than 25 μg and 50 μg for preventing PONV in nonobese female patients. Our study demonstrated that this dose of 75 μg is also effective for obese female patients with a BMI ≥ 30 kg m^-2^.

Another study [13] that compared three weight-adjusted doses of palonosetron (0.5, 1.0, or 1.5 μg kg^-1^) but had as an exclusion criteria a BMI ≥ 30 kg m^-2^ found a significant dose-dependent decrease in the incidence of PONV. In our study of obese patients, increasing doses of palonosetron did not show a significant difference in PONV incidence between the two groups. These considerations suggest that doses of palonosetron greater than 75 μg are not able to improve its effectiveness in preventing PONV. Therefore, a fixed dose may be preferable to a body weight-adjusted dose, since it may be more cost-effective in obese patients, as a lower dose can be used for the same effectiveness in PONV prevention.

Further studies using higher body weight-adjusted doses or patients with higher total body weight are needed to evaluate the dose-dependent effect of palonosetron, since in the present study, the difference between the doses used was not statistically significant (P = 0.164).

The limitations of the present study are as follows: 1 –A placebo group was not included because it was considered unethical to withhold prophylactic antiemetic drugs from patients at risk for PONV. 2 –Combination therapy with palonosetron and dexamethasone was necessary because the recruited group had high-risk Apfel scores. 3 –Although younger age (< 50 years) has been found to be a risk factor for PONV in the literature [1], the population of this study included a broader age range (18–80 years).

## Conclusions

A body weight-adjusted dose of palonosetron at 1 μg kg^-1^ was as effective as a fixed dose of 75 μg for preventing PONV in the first 48 h postoperatively among obese female patients undergoing breast surgery. Hence, the fixed dose may be preferable to the body weight-adjusted dose.

## Supporting information

S1 CONSORT Checklist CONSORT 2010 checklist of information to include when reporting a randomised trial. (DOC)

S1 Protocol Study protocol—Original version submitted /approved by ethics committee. (DOCX)

S2 Protocol Study protocol English—Translation of the main points. (DOCX)

S1 Data Patient´s data. (XLSX)
