# Supplementary material for: A prospective, randomized, double-blind trial to compare body weight-adjusted and fixed doses of palonosetron for preventing postoperative nausea and vomiting in obese female patients
Source: PLoS One. 2020 Jan 14;15(1):e0227490. doi: 10.1371/journal.pone.0227490 (PMC6959980; doi:10.1371/journal.pone.0227490)
Supplement: S1 Protocol — (DOCX) [file pone.0227490.s002.docx]

Linha de Pesquisa: Fármacos em Procedimentos Anestésico-cirúrgicos

**ESTUDO COMPARATIVO ENTRE DUAS DOSES DE PALONOSETRONA NA PROFILAXIA DE NÁUSEAS E VÔMITOS PÓS-OPERATÓRIOS EM PACIENTES OBESAS SUBMETIDAS À CIRURGIA DE MAMA**

PROTOCOLO DE PESQUISA

**Pesquisador:** Nathalia Gouveia de Araújo Ferreira

**Orientador:** Professora Doutora Nubia Verçosa Figueiredo

**Co-orientador:** Professor Doutor Ismar Lima Cavalcanti

**Rio de Janeiro/RJ**

**2016**

**SUMÁRIO**

**1. Introdução.....................................................................................................3**

**2. Hipótese........................................................................................................4**

**3. Objetivos.......................................................................................................4**

**4. Justificativas.................................................................................................5**

**5. Análise crítica de riscos e benefícios.........................................................5**

**6. Seleção, aleatorização e cegamento dos pacientes.................................6**

**7. Critérios de inclusão....................................................................................6**

**8. Critérios de exclusão...................................................................................7**

**09. Métodos.......................................................................................................7**

**10. Registro e apresentação de dados..........................................................10**

**11. Método do cálculo do tamanho da amostra e poder do estudo...........11**

**12. Análise estatística......................................................................................11**

**13. Cronograma (a partir da aprovação)........................................................12**

**14. Responsabilidades....................................................................................12**

**15. Critérios para suspender ou encerrar a pesquisa..................................14**

**16. Infraestrutura.............................................................................................14**

**17. Propriedade das informações geradas pela pesquisa..........................14**

**18. Curriculum Lattes..........................................................................................15**

**19. Referências.....................................................................................................15**

1. **Introdução**

A estratégia visando à profilaxia de náuseas e vômitos pós-operatórios é uma rotina em todo procedimento anestésico-cirúrgico realizado, já que o desconforto e a morbidade associados a essa complicação anestésica são amplamente conhecidos. Os antagonistas serotoninérgicos já são uma classe de medicação amplamente utilizada para esse fim e que apresentam eficácia superior em relação aos outros antieméticos^1^. Seu mecanismo de ação consiste na inibição do influxo de cálcio causado pela estimulação dos receptores do subtipo 5-HT_3_ nos neurônios localizados na área postrema (zona do gatilho emético)^2,6^.

A palonosetrona é um antagonista serotoninérgico de segunda geração com afinidade elevada de ligação ao receptor 5-HT_3_. Tem uma estrutura química única e com maior meia vida plasmática que os demais medicamentos dessa classe^3,4^. Foi inicialmente estudado como antiemético pós-quimioterapia^5^, mas rapidamente também se mostrou eficaz na profilaxia de náuseas e vômitos pós-operatórios (PNVPO)^6^. A Palonosetrona é normalmente utilizada em uma dose fixa de 75 mcg para a PNVPO, apesar de alguns estudos terem sido realizados utilizando a dose ajustada pelo peso^7,8^, ainda é necessária a determinação da dose ideal para pacientes obesos, uma vez que, até a presente data não há estudo clínico publicado nesse grupo populacional.

1. **Hipótese**

A hipótese do presente estudo é que o uso da palonosetrona em uma dose ajustada pelo peso (1mcg/kg) é mais eficaz que uma dose fixa (75mcg) na prevenção de náuseas e vômitos pós-operatórios nas cirurgias de mama em pacientes do gênero feminino com índice de massa corporal igual ou acima de 30 kg/m^2^.

**3. Objetivos**

**Primários:**

3.a) Determinar a frequência dos episódios individuais de náuseas no período de 1, 6, 24 e 48 horas no pós-operatório;.
3.b) Determinar a frequência dos episódios individuais de vômitos no período de 1, 6, 24 e 48 horas no pós-operatório;

3.c) Comparar a frequência da necessidade de uso de medicação de resgate antiemética nos períodos de 1, 6, 24 e 48 horas no pós-operatório;

3.d) Determinar o número de pacientes que apresentaram resposta antiemética completa (ausência de náusea, de vômitos e de necessidade de medicação de resgate).

**Secundário:**

Avaliar a frequência do polimorfismo dos receptores 5-HT_3_ e a sua correlação com a intensidade da ação antiemética e dos efeitos adversos

**4. Justificativa**

A palonosetrona, um antagonista dos receptores de serotonina 5-HT_3_ de segunda geração, tem sido crescentemente utilizado na profilaxia de náuseas e vômitos pós-operatório. Entretanto, a maioria dos estudos utiliza uma dose fixa de 75mcg de palonosetrona intravenoso, sendo necessária uma avaliação do benefício da utilização de uma dose ajustada pelo peso corporal na determinação da eficácia do fármaco em pacientes femininas e obesas. Até a presente data não há estudo clínico publicado nesse grupo populacional.

Serão incluídas as avalições dos polimorfismos genético dos receptores 5-HT_3_ para avaliar a frequência dessa alteração e sua influência na ação antiemética.

**5. Análise crítica de riscos e benefícios**

As ocorrências de náuseas e vômitos no pós-operatório preocupam o anestesiologista porque elas acontecem frequentemente, propiciam o retardo na recuperação cirúrgica e reduzem o grau de satisfação do paciente. Os benefícios da profilaxia de náuseas e vômitos no pós-operatório (PNVPO) já foram amplamente estudados. Os antagonistas 5-HT_3_ têm eficiência reconhecida e apresentam-se como terapia de primeira linha. A determinação da melhor dose para o uso da palonosetrona poderá aumentar a eficácia do seu uso na PNVPO.

Alguns efeitos adversos podem ocorrer no presente estudo e os mais frequentes são: sedação, cefaleia e vertigem. Existem, porém, os riscos inerentes aos procedimentos anestésico-cirúrgicos, tais como: infecções, hemorragias, arritmias, alergias e dor, entre outros.

**6. Seleção, aleatorização e cegamento dos pacientes**

As pacientes selecionadas serão do gênero feminino, com índice de massa corporal igual ou maior que 30 kg/m^2^, que serão submetidas às cirurgias de mama (mastectomia simples, mastectomia radical modificada e ressecção segmentar de mama). Todos os procedimentos serão realizados no Instituto Nacional do Câncer (INCA), unidade hospitalar III (HCIII).

As pacientes serão distribuídas de forma aleatória em dois grupos: um com dose fixa de 75 mcg de palonosetrona intravenosa e outro com dose ajustada pelo peso (1mcg/kg) de palonosetrona também por via endovenosa. A aleatorização será feita eletronicamente. Por se tratar de estudo duplamente encoberto, tanto o pesquisador quanto o paciente não saberão qual dose de fármaco será administrada.

**7. Critérios de inclusão:**

7.a) Gênero feminino;

7.b) Idade entre 18 e 70 anos;

7.c) Estado físico segundo a *American Society of Anesthesiologists* (ASA): I, II, III;
7.d) Cirurgias de mama (mastectomia simples, mastectomia radical modificada e ressecção segmentar de mama)
7.e) Índice de massa corporal igual ou maior que 30 kg/m^2^

**8. Critérios de exclusão:**

8.a.) Ocorrência de episódios de náusea ou vômitos nas últimas 24h que antecedem á cirurgia;

8.b.) uso de corticosteroides;

8.c.) tabagismo;

8.d.) alcoolismo;

8.e.) uso de fármacos psicoativos ou qualquer outro medicamento com efeito antiemético;

8.f) hipersensibilidade à medicação estudada;
8.g) cirurgias em caráter de urgência e emergência;
8.h) quimioterapia há menos de 4 semanas.

**9. Métodos**

O ensaio clínico, prospectivo, aleatório e duplamente encoberto será realizado no Instituto Nacional do Câncer (INCA), unidade III (HCIII), Rio de Janeiro situado na Rua Visconde de Santa Isabel, 274, Vila Isabel, Rio de Janeiro, RJ.

O Termo de Consentimento Livre e Esclarecido (TCLE) será apresentado e assinado, antes da cirurgia, por cada participante voluntária, as quais serão orientadas acerca dos riscos e benefícios da pesquisa, segundo resolução CNS 466/12.

As pacientes serão distribuídas aleatoriamente em dois grupos: o grupo I

(GI) receberá uma dose ajustada pelo peso corporal de 1mcg/kg de palonosetrona intravenoso e o Grupo II (GII), receberá uma dose fixa de palonosetrona de 75 mcg intravenoso. A palonosetrona, não é uma medicação padronizada no INCA, será, portanto, doada à farmácia do INCA pelo pesquisador, obedecendo às normas vigentes da instituição hospitalar.

As pacientes serão avaliadas clinicamente no Ambulatório de Avaliação Pré-Anestésica. No dia da cirurgia, na enfermaria, receberão medicação pré-anestésica (midazolam 15 mg via oral). Na sala de cirurgia, após a venóclise, deverá ser iniciada a monitorização básica (pressão arterial não invasiva, cardioscopia, oximetria de pulso), colocação da máscara laríngea, do capnógrafo, posicionamento do índice bispectral (BIS) e do estimulador de nervo periférico. A palonosetrona será administrada um minuto antes da indução anestésica, em *bolus*, por via intravenosa. A dose determinada da medicação será diluída em uma seringa de 10ml com água destilada, por um anestesiologista não envolvido na pesquisa, permitindo que, o pesquisador fique encoberto para a dose utilizada.

As pacientes serão pré-oxigenadas e a indução anestésica será realizada com administração intravenosa de fentanil 3 mcg/kg, lidocaína 1,5 mg/kg, propofol 1,5 mg/kg e rocurônio 0,3mg/kg. Em seguida, será realizada a inserção da máscara laríngea adequada para permitir ventilação pulmonar com 6ml/kg de peso ideal e pressão de pico máxima 25 cmH_2_O. Na manutenção da anestesia o sevoflurano será utilizado, com um fluxo de 2 L/min e 50% oxigênio/ar, com sua concentração ajustada para manter BIS entre 40-60. O remifentanil na dose de 0,05 mcg/kg/min a 0,2 mcg/kg/min, por via intravenosa, poderá ser administrado, no intraoperatório, caso a frequência cardíaca ou a pressão arterial se elevem acima de 20% dos valores basais. Doses adicionais de rocurônio também poderão ser administradas conforme a necessidade. O bloqueio neuromuscular será revertido com sugamadex (2mg/kg) no momento em que recuperarem duas respostas ao monitor de função neuromuscular, e será anotado o período de tempo (em segundos) para que haja a recuperação de 90% de resposta muscular. Para analgesia pós-operatória, as pacientes receberão tenoxican 40 mg, dipirona 50 mg/kg e morfina 2 mg intravenoso 40 minutos antes do término da cirurgia. As pacientes serão então, encaminhadas à sala de recuperação pós-anestésica (RPA), onde serão observadas por 2 horas e em seguida encaminhadas à enfermaria. A alta hospitalar estará prevista para 24 horas após o procedimento cirúrgico.

Ambos os grupos receberão o antiemético metoclopramida 10mg, por via intravenosa, como medicação de resgate para NVPO nas primeiras 24h de pós-operatório.

As pacientes serão questionadas após 1, 6, 24 e 48 horas de pós-operatório quanto à frequência de náuseas ou vômitos e será avaliada a frequência da necessidade de utilização da medicação de regate nesses períodos. Aquelas pacientes que receberem alta hospitalar após 24 horas receberão uma ligação telefônica para a avaliação do período até 48 horas de pós-operatório.

Para o propósito do estudo, a náusea será definida como sensação desagradável e involuntária de vomitar, sem a expulsão de conteúdo gástrico e vômito como expulsão do conteúdo gástrico.

Também será avaliada a frequência e a quantidade de utilização de opioides como terapia de resgate para analgesia pós-operatória.

Os resultados das análises dos polimorfismos também serão analisados.

As pacientes terão amostras celulares da região vestibular da cavidade oral, coletadas através de esfregaço, analisadas no laboratório da Unidade de Pesquisa Clínica da Universidade Federal Fluminense (UFF), para pesquisa concomitante de prevalência de polimorfismo dos receptores 5-HT_3_ e sua correlação com o efeito antiemético. O material coletado será colocado em um tubo e em seguida será acomodado no congelador do laboratório do hospital. O pesquisador principal transportará as amostras da cavidade oral semanalmente, do laboratório do INCA 3 para o Laboratório de Pesquisa Clínica da UFF, em caixa específica para transporte de material biológico, contendo gelo e termômetro para que possa ser mantida a refrigeração adequada. Seguindo normas de controle de qualidade, serão excluídas da análise de polimorfismo 5-HT_3_ as amostras que demorarem mais de sete dias para serem analisadas a partir da data da coleta.

O DNA será extraído a partir de amostras de células da cavidade oral utilizando um kit de purificação de DNA genômico. Os testes, de reação de polimerase em cadeia (PCR) em tempo real, serão aplicados para análise dos polimorfismos de nucleotídeos simples. As reações serão preparadas com genotipagem de acordo com as instruções do fabricante e realizadas sobre um sistema de detecção de sequência, utilizando condições de ciclos térmicos padrão. As amostras biológicas serão descartadas no final da análise.

**10. Registro e apresentação de dados**

Os dados gerados pela pesquisa serão registrados em questionário específico que deverá ser preenchido pelo pesquisador com os dados obtidos de cada paciente. Essas informações serão apresentadas e servirão de base para análise estatística do trabalho.

**11. Método do cálculo do tamanho da amostra e poder do estudo**

O tamanho da amostra foi calculado tendo como base o desfecho primário. Utilizou-se como parâmetro resultado anteriormente publicado que demonstrou uma redução da ordem de 30% na incidência de náuseas e vômitos pós-operatórios, quando se utilizou profilaxia farmacológica para náuseas e vômitos. Uma amostra de 35 pacientes em cada grupo é necessária para detectar tal diferença, com probabilidade de erro tipo-1 de 0,05 (α) e de erro tipo-2 de 0,2 (β) pelo teste de duas caudas (poder do estudo de 80%). Quarenta pacientes, em cada grupo, serão recrutados devido a possíveis perdas de seguimento.

**12. Análise estatística**

Todas as análises estatísticas serão realizadas pelo programa GraphPad Prism 4.0® (GraphPad Softwares Inc., San Diego, CA, USA). O Teste de Wilcoxon rank 2x2 será utilizado para avaliar a distribuição normal dos dados. As características dos pacientes e as variáveis estudadas no momento pós-operatório serão analisadas pelo teste T Student´s. Os dados categóricos serão comparados utilizando o teste de Qui Quadrado ou teste exato de Fisher's. O valor de P<0,05 será considerado significante estatisticamente

**13. Cronograma (a partir da aprovação)**

Com o início da fase de coleta de dados em junho/2016, a duração da pesquisa deverá ser de 12 (doze) meses, podendo se estender por mais 6 (seis) meses. A fase de coleta de dados deverá ser encerrada ao término do primeiro semestre de 2018. Após a finalização da análise estatística, deverá ser iniciada a redação de um artigo científico e enviado para publicação em uma revista indexada internacional.

**14. Responsabilidades**

**14.a) Do pesquisador**

**Responsáveis pelo ensaio / pesquisadores principais**

**Nome**: Nathalia Gouveia de Araújo Ferreira, MD.

**Unidade:** Serviço de Anestesiologia do Instituto Nacional do Câncer

**Categoria funcional / regime de trabalho:** Médico Anestesiologista; 40 horas/semana.

**Responsabilidades:** Seleção dos candidatos, administração da anestesia e coleta e analise dos dados.

**Orientador**

**Nome:** Nubia Verçosa Figueiredo, MD, PhD.

**Unidade:** Departamento de Cirurgia - UFRJ

**Categoria funcional / regime de trabalho:** Professora Associada IV. **Responsabilidades:** elaboração do projeto de pesquisa; processamento dos dados; análise estatística.

**Co-orientador**

**Nome**: Ismar Lima Cavalcanti, MD, PhD.

**Unidade:** Departamento de Cirurgia Geral e Especializada - UFF e Departamento de Cirurgia - UFRJ

**Categoria funcional / regime de trabalho:** Professor Adjunto II e Professor Colaborador

**Responsabilidades:** elaboração do projeto de pesquisa; coordenação da pesquisa, processamento dos dados; análise estatística.

**Co-autores**

**Nome**: Alexandra Rezende Assad, MD, PhD.

**Unidade:** Departamento de Cirurgia Geral e Especializada - UFF

**Categoria funcional / regime de trabalho:** Professor

**Responsabilidades:** elaboração do projeto de pesquisa; processamento dos dados, redação do artigo.

**Nome**: Estevão Braga, MD, PhD.

**Unidade:** Departamento de Cirurgia Geral e Especializada - UFF

**Categoria funcional / regime de trabalho:** Professor

**Responsabilidades:** elaboração do projeto de pesquisa; processamento dos dados.

**Nome**: Louis Barrucand, MD, PhD.

**Unidade:** Faculdade Medicina universidade federal do rio de janeiro - UFRJ

**Categoria funcional / regime de trabalho:** Professor

**Responsabilidades:** processamento dos dados; análise estatística.

**14.b) Da instituição**

Oferecer condições dignas e apropriadas para a execução das consultas ambulatoriais, internação, ato e recuperação anestésico-cirúrgico no Instituto Nacional do Câncer, Unidade Hospitalar III (HCIII)

**14.c) Do patrocinador e conflito de interesses**

O presente estudo não possui patrocinador.

Não tem conflito de interesses.

**15. Critérios para suspender ou encerrar a pesquisa**

O ensaio será suspenso, em qualquer ocasião, caso surjam eventos adversos inesperados que justifiquem a suspensão do trabalho. Neste caso, será comunicado à Comissão de Ética local e ao CONEP.

**16. Infraestrutura**

**16.a) local da pesquisa**

O ato anestésico cirúrgico e a coleta de dados serão desenvolvidos no Instituto Nacional do Câncer, Unidade Hospitalar III, no endereço: Rua Visconde de Santa Isabel, 274, Vila Isabel, Rio de Janeiro, RJ, Serviço de Anestesiologia. Segundo normas do CFM (Resolução n^º^1.802/96), o procedimento anestésico cirúrgico ocorrerá em ambiente apropriado, em sala com saída de gases, aparelho de anestesia, monitorização continua e equipamentos de reanimação cardiopulmonar. As internações hospitalares ocorrerão na enfermaria da Mastologia, com a recuperação e liberação do paciente ocorrendo no mínimo após 24h de pós-operatório, após avaliação clínica.

**16.b) Orçamento financeiro**

Toda a estrutura necessária ao desenvolvimento da pesquisa já existe no INCA III. A pesquisa não onerará a instituição com outros gastos além dos previstos. As despesas com material de consumo (papel, impressão de fichas clínicas e de evolução) serão financiadas pelo pesquisador responsável.

**17. Propriedade das informações geradas pela pesquisa**

**17.a) Fontes de material de pesquisa**

Funcionarão como fonte de material e pesquisa o resultado de exames, a ficha de avaliação clínica e o prontuário dos pacientes.

**17.b) Uso e destinação do material e dados coletados**

Os dados coletados destinam-se exclusivamente aos fins especificados no presente projeto.

**17.c) Publicação dos resultados**

Os resultados do projeto de pesquisa “**Estudo comparativo entre duas doses de palonosetrona na profilaxia de náuseas e vômitos pós-operatórios em pacientes obesas submetidas à cirurgia de mama”** serão tornados públicos, sejam eles favoráveis ou não. Detêm a propriedade intelectual das informações geradas pelo ensaio os pesquisadores

responsáveis e os demais participantes do projeto.

**18. Curriculum Lattes**

Nathalia Gouveia de Araujo Ferreira

http://lattes.cnpq.br/8629863615107046

Nubia Verçosa Figueiredo

http://lattes.cnpq.br/7092225148739715

Ismar Lima Cavalcanti

<http://lattes.cnpq.br/3897507589015450>

**QUESTIONÁRIO DE COLETA DE DADOS**

**Número da aleatorização:______**

**Idade:______ Peso:______ Altura:______ IMC:______**

**ASA:______**

**Cirurgia:________________________________________________________**

**Tempo cirúrgico total:______ min**

**Fatores de risco:**

**( ) História prévia NVPO ( ) História prévia cinetose**

| **Intervalo 0 - 1h pós-operatório** |
| --- |
| **Analgesia de resgate com opioide:**  **Dose:**  **Intervalo de administração:** |
| **Antiemético de regaste:**  **Dose:**  **Intervalo de administração:** |
| **Presença náusea: episódios** |
| **Presença vômitos: episódios** |

| **Intervalo 1 - 6h pós-operatório** |
| --- |
| **Analgesia de resgate com opioide:**  **Dose:**  **Intervalo de administração:** |
| **Antiemético de regaste:**  **Dose:**  **Intervalo de administração:** |
| **Presença náusea: episódios** |
| **Presença vômitos: episódios** |

| **Intervalo 6 - 24h pós-operatório** |
| --- |
| **Analgesia de resgate com opióide:**  **Dose:**  **Intervalo de administração:** |
| **Antiemético de regaste:**  **Dose:**  **Intervalo de administração:** |
| **Presença náusea: episódios** |
| **Presença vômitos: episódios** |

| **Intervalo 24 - 48h pós-operatório** |
| --- |
| **Analgesia de resgate com opioide:**  **Dose:**  **Intervalo administração:** |
| **Antiemético de regaste:**  **Dose:**  **Intervalo de administração:** |
| **Presença náusea: episódios** |
| **Presença vômitos: episódios** |

| **Indução anestésica - Doses** |  |
| --- | --- |
| **Propofol:** | **Rocurônio:** |
| **Fentanil:** | **Remifentanil:** |
| **Lidocaína:** | **Tempo reversão BNM:** |

TERMO DE CONSENTIMENTO LIVRE E ESCLARECIDO

***Estudo comparativo entre duas doses de palonosetrona na profilaxia de náuseas e vômitos pós-operatórios em pacientes obesas submetidas à cirurgia de mama***

Você está sendo convidado (a) a participar de uma pesquisa porque foi atendido (a) ou esta sendo atendido (a) nesta instituição e teve diagnóstico ou suspeita de um tipo de câncer de mama. Para que você possa decidir se quer participar ou não, precisa conhecer os benefícios, os riscos e as consequências pela sua participação. Este documento é chamado de Termo de Consentimento Livre e Esclarecido e tem esse nome porque você só deve aceitar participar desta pesquisa depois de ter lido e entendido este documento. Leia as informações com atenção e converse com o pesquisador responsável e com a equipe da pesquisa sobre quaisquer dúvidas que você tenha. Caso haja alguma palavra ou frase que você não entenda, converse com a pessoa responsável por obter este consentimento, para maiores esclarecimentos. Converse com os seus familiares, amigos e com a equipe médica antes de tomar uma decisão. Se você tiver dúvidas depois de ler estas informações, entre em contato com o pesquisador responsável. Após receber todas as informações, e todas as dúvidas forem esclarecidas, você poderá fornecer seu consentimento por escrito, caso queira participar.

#### PROPÓSITO DA PESQUISA

1. O propósito dessa pesquisa é determinar qual a dose ideal da medicação chamada Palonosetrona para evitar náusea e vômitos após procedimento cirúrgico em pacientes obesas submetidas à cirurgia na mama

#### PROCEDIMENTOS DA PESQUISA

Na ocasião da cirurgia, e só após a sua autorização, você receberá uma dose da medicação chamada Palonosetrona. Você será incluído, de forma aleatória (ou seja não poderá ser escolhido por você, nem pelo pesquisador), em um dos dois grupos do estudo: Grupo I receberá a dose da medicação de 75 microgramas e Grupo II receberá a dose da medicação de 1 micrograma a cada kilo de peso corporal. Você não saberá em qual grupo foi incluído, pois isso é importante para que os dados do estudo sejam verdadeiros; Após a cirurgia será perguntada em três diferentes ocasiões (6, 24 e 48 horas após a cirurgia), em visita no hospital ou por contato telefônico, se apresentou náusea ou vômitos. Na ocasião da cirurgia, e só após sua autorização, uma amostra de células de esfregaço da sua boca (raspagem da parte interna da bochecha) será coletada para análise genética do local de ação da medicação. Este procedimento tem como objetivo procurar uma explicação genética para a diferença de eficácia da medicação em diferentes indivíduos. Todas as amostras biológicas coletadas durante esta pesquisa, conforme descrito acima, serão utilizadas apenas para os propósitos descritos neste Termo de Consentimento Livre e Esclarecido. Ao final da pesquisa ou depois que todos os resultados dos exames ficarem prontos, se tiver sobrado alguma quantidade de sangue ou células de esfregaço da cavidade oral, estas amostras serão destruídas. O laboratório não irá guardar suas amostras biológicas.

**MÉTODOS ALTERNATIVOS**

Caso você apresente náusea ou vômitos mesmo após o uso da medicação pesquisada, será dado outro tipo de medicamento para o tratamento dessas náuseas e vômitos.

**BENEFÍCIOS**

Você não será remunerado por sua participação e esta pesquisa poderá não oferecer benefícios diretos a você. Se você concordar com o uso de suas informações e/ou do material do modo descrito acima, é necessário esclarecer que você não terá quaisquer benefícios ou direitos financeiros sobre eventuais resultados decorrentes desta pesquisa.

O benefício principal da sua participação é possibilitar no futuro, com os resultados alcançados com esta pesquisa, a melhora do tratamento para náusea e vômitos após procedimentos cirúrgicos beneficiem outros pacientes.

**RISCOS**

Não existem riscos físicos adicionais a você pela sua participação nesta pesquisa. Os riscos físicos e inconvenientes não serão diferentes daqueles previstos durante os procedimentos normais para realização da sua cirurgia.

Alguns efeitos adversos podem ocorrer com o uso da medicação, sendo os mais frequentes: sedação, cefaleia e vertigem. Existem, também, os riscos inerentes aos procedimentos anestésico-cirúrgicos, tais como: infecções, hemorragias, arritmias, alergias e dor.

**CUSTOS**

Se você concordar com essa pesquisa, você não terá quaisquer custos ou despesas (gastos) pela sua participação nessa pesquisa. Você não pagará por qualquer procedimento, medicação em estudo ou teste exigido como parte desta pesquisa.

**CONFIDENCIALIDADE**

Se você optar por participar desta pesquisa, as informações sobre a sua saúde e seus dados pessoais serão mantidas de maneira confidencial e sigilosa. Seus dados somente serão utilizados depois de anonimizados (ou seja, sem sua identificação). Apenas os pesquisadores autorizados terão acesso aos dados individuais, resultados de exames e testes bem como às informações do seu registro médico. Mesmo que estes dados sejam utilizados para propósitos de divulgação e/ou publicação científica, sua identidade permanecerá em segredo.

______________________________ _____________________

Rubrica do participante da pesquisa Rubrica do pesquisador

**TRATAMENTO MÉDICO EM CASO DE DANOS**

Todo e qualquer dano decorrente do desenvolvimento desta pesquisa, e que necessite de atendimento médico, ficará a cargo da instituição. Seu tratamento e acompanhamento médico independem de sua participação nesta pesquisa.

**BASES DA PARTICIPAÇÃO**

A sua participação é voluntária e a recusa em autorizar a sua participação não acarretará quaisquer penalidades ou perda de benefícios aos quais você tem direito, ou mudança no seu tratamento e acompanhamento médico nesta instituição. Você poderá retirar seu consentimento a qualquer momento sem qualquer prejuízo. Em caso de você decidir interromper sua participação na pesquisa, a equipe de pesquisadores deve ser comunicada e a pesquisa será imediatamente interrompida.

**ACESSO AOS RESULTADOS DA PESQUISA**

Você pode ter acesso a qualquer resultado relacionado à esta pesquisa. Se você tiver interesse, você poderá receber uma cópia dos mesmos.

**GARANTIA DE ESCLARECIMENTOS**

A pessoa responsável pela obtenção deste Termo de Consentimento Livre e Esclarecido lhe explicou claramente o conteúdo destas informações e se colocou à disposição para responder às suas perguntas sempre que tiver novas dúvidas. Você terá garantia de acesso, em qualquer etapa da pesquisa, sobre qualquer esclarecimento de eventuais dúvidas e inclusive para tomar conhecimento dos resultados desta pesquisa. Neste caso, por favor, ligue para o(a) Nathalia Gouveia de Araujo Ferreira, no telefone (21) 996445380 de 8:00 as18:00 hs. Esta pesquisa foi aprovada pelo Comitê de Ética em Pesquisa (CEP) do INCA, que está formado por profissionais de diferentes áreas, que revisam os projetos de pesquisa que envolvem seres humanos, para garantir os direitos, a segurança e o bem-estar de todos as pessoas que se voluntariam à participar destes. Se tiver perguntas sobre seus direitos como participante de pesquisa, você pode entrar em contato com o CEP do INCA na Rua do Resende N°128, Sala 203, de segunda a sexta de 9:00 a 17:00 hs, nos telefones (21) 3207-4550 ou 3207-4556, ou também pelo e-mail: cep@inca.gov.br.

Este termo está sendo elaborado em duas vias, sendo que uma via ficará com você e outra será arquivada com os pesquisadores responsáveis.

**CONSENTIMENTO**

Li as informações acima e entendi o propósito da solicitação de permissão para o uso das informações contidas no meu registro médico e das células de esfregaço da minha boca obtidas durante o atendimento nesse hospital. Tive a oportunidade de fazer perguntas e todas foram respondidas. Ficaram claros para mim quais são procedimentos a serem realizados, riscos e a garantia de esclarecimentos permanentes. Ficou claro também que a minha participação é isenta de despesas e que tenho garantia do acesso aos dados e de esclarecer minhas dúvidas a qualquer tempo. Entendo que meu nome não será publicado e toda tentativa será feita para assegurar o meu anonimato. Concordo voluntariamente em participar desta pesquisa e poderei retirar o meu consentimento a qualquer momento, antes ou durante o mesmo, sem penalidade ou prejuízo ou perda de qualquer benefício que eu possa ter adquirido.

Eu, por intermédio deste, dou livremente meu consentimento para participar nesta pesquisa.

|  |  | / / |
| --- | --- | --- |
| Nome e Assinatura do participante |  | Data |
|  |  |  |
|  |  | / / |
| Nome e Assinatura do Responsável Legal/Testemunha Imparcial  (Quando pertinente) |  | Data |

Eu, abaixo assinado, expliquei completamente os detalhes relevantes desta pesquisa ao paciente indicado acima e/ou pessoa autorizada para consentir pelo mesmo. Declaro que obtive de forma apropriada e voluntária o Consentimento Livre e Esclarecido deste paciente para a participação desta pesquisa.

|  |  | / / |
| --- | --- | --- |
| Nome e Assinatura do Responsável pela obtenção do Termo |  | Data |
