# Supplementary material for: A prospective, randomized, double-blind trial to compare body weight-adjusted and fixed doses of palonosetron for preventing postoperative nausea and vomiting in obese female patients
Source: PLoS One. 2020 Jan 14;15(1):e0227490. doi: 10.1371/journal.pone.0227490 (PMC6959980; doi:10.1371/journal.pone.0227490)
Supplement: S2 Protocol — (DOCX) [file pone.0227490.s003.docx]

### COMPARATIVE STUDY BETWEEN TWO DOSES OF PALONOSETRON ON THE PREVENTION OF POSTOPERATIVE NAUSEA AND VOMITING IN OBESE PATIENTS UNDERGOING BREAST SURGERY

### Investigators/Researchers:

Nathalia Gouveia de Araújo Ferreira, M.D., Anesthesiologist. MSc student of Department of Anesthesiology of Federal University of Rio de Janeiro, Brazil.

### Nubia Verçosa Figueiredo, M.D., Professor of Department of Anesthesiology of Federal University of Rio de Janeiro, Brazil.

Ismar Lima Cavalcanti, M.D., Professor of Department of Anesthesiology of Federal Fluminense University, Rio de Janeiro, Brazil.

Alexandra Rezende Assad, M.D., Professor of Department of Anesthesiology of Federal Fluminense University, Rio de Janeiro, Brazil

Louis Barrucand, M.D., Professor Faculty of Medicine, Federal University of Rio de Janeiro, Rio de janeiro, Brazil

Estevão Braga, M.D., Professor of Department of Anesthesiology of Federal Fluminense University, Rio de Janeiro, Brazil

**Abstract/Summary:**

BACKGROUND: Palonosetron is a second generation serotonin antagonist usually used in a ﬁxed dose of 75 mcg for the prophylaxis of postoperative nausea and vomiting. Although there are a few trials evaluating bodyweight-adjusted doses of palonosetron, the ideal dose for obese patients has not yet been investigated. The aim of this study is to evaluate if the use of bodyweight-adjusted doses of palonosetron (1mcg / kg) is more effective than a fixed dose (75mcg) on preventing postoperative nausea and vomiting in female patients with a body mass index equal to or above 30 kg /m2 whom have undergone breast surgery

METHODS: A prospective, randomized double-blind trial will be conducted at the National Cancer Institute (INCA - HCIII), in Rio de Janeiro, Brazil. Eligible patients are female aged between 18-70 years of age with an ASA physical status of one to three, with body mass index equal to or greater than 30 kg / m2, scheduled to undergo elective breast surgery. Forty patients will be randomized to receive a bodyweight-adjusted dose of 1mcg/kg of palonosetron (group I - GI) and 40 patients will receive a fix dose of 75 μg of palonosetron (group II - GII). Patients will be assessed over 4 time periods: 0-1h, 1-6 h, 6-24 h and 24-48 h after surgery. After discharge, all patients will be contacted by telephone at regular intervals to record the episodes of nausea and vomiting as well as analgesic and antiemetic requirements. The primary outcome measure will include the number of emetic episodes, the incidence of nausea, the need for rescue antiemetics and the analgesic requirement over 48h. The secondary outcome will be to evaluate the frequency of polymorphism of the 5-HT3 receptors and their correlation with the antiemetic effect and adverse effects.

**Introduction**

Prevention of nausea and vomiting after surgery is a routine throughout anesthetic-surgical procedure, since the discomfort and morbidity associated with this anesthetic complication are widely known. The serotonin antagonists are largely used for this purpose because they exhibit superior prophylactic efficacy when compared with other antiemetic drugs. Its mechanism of action is the inhibition of calcium influx caused by stimulation of serotonin 5-hydroxytryptamine type 3 receptor (5-HT3).

Palonosetron is a second generation serotonin antagonist with high affinity binding to the 5-HT3 receptor. It has a unique chemical structure and longer half-life than older 5-HT3 antagonists. It was initially approved for use in the prevention of chemotherapy induced nausea and vomiting but also proved effective in the prophylaxis of postoperative nausea and vomiting. Palonosetron is usually used in a ﬁxed dose of 75 mcg for the prophylaxis of postoperative nausea and vomiting. Although there are a few trials evaluating bodyweight-adjusted doses of palonosetron, the ideal dose of obese patient has not yet been investigated.

**Aims & Objectives**

Our hypothesis for this study is that the use of bodyweight-adjusted doses of palonosetron (1mcg / kg) is more effective than a fixed dose (75mcg) for preventing postoperative nausea and vomiting in female patients with a body mass index equal to or above 30 kg / m2 whom have undergone breast surgery.

Primary objectives:

1. To determine the frequency of individual episodes of nausea in the periods of 1, 6, 24 and 48 hours postoperatively
2. To determine the frequency of individual episodes of vomiting in the periods of 1, 6, 24 and 48 hours postoperatively
3. Compare need of antiemetic rescue medication during the periods of 1, 6, 24 and 48 hours postoperatively
4. Determine the number of patients who had complete antiemetic response (absence of nausea, vomiting and need for rescue medication).

Secondary objectives: To evaluate the frequency of polymorphism of the 5-HT3 receptors and their correlation with the antiemetic effect and adverse effects

### Methods

A prospective, randomized double-blind trial will be conducted at the National Cancer Institute (INCA - HCIII), in Rio de Janeiro, Brazil. All patients will be provided with written informed consent form prior to study enrolment. Eligible patients are female aged between 18-70 years of age with an ASA physical status of one to three, with body mass index equal to or greater than 30 kg / m2, scheduled to undergo elective breast surgery. The exclusion criteria are: patients who had received chemotherapy within 4 weeks, patients who have undergone emergency surgery, with known history of vomiting within 24 hours; a known hypersensitivity to other 5-HTantagonists, patients who received any antiemetic, corticosteroid, or other drug with antiemetic effect, smokers and patients with a history of alcoholism.

Using computer-generated random numbers, the patients will be assigned into one of the two treatment groups on the morning of surgery. Forty patients will be randomized to receive a bodyweight-adjusted dose of 1mcg/kg of palonosetron (group I - GI) and another forty patients will receive a fix dose of 75 μg of palonosetron (group II - GII). Each study drug will be mixed with saline to a total volume of 10 ml in an unlabelled syringe and will be intravenously administered just prior to induction of anesthesia. All patients, surgeons, anesthesiologists involved in the study will be blinded to group allocation to maintain the double-blind conditions.

All patients will receive premedication with midazolam. Intraoperative monitoring included electrocardiography, blood pressure measurement, peripheral oxygen saturation (SpO2), and end-tidal CO2 tension (ETco2). General anesthesia was induced using 1,5 mg/kg of propofol, 3 μg/kg of fentanyl, 1,5 mg/kg of lidocaine and 0.3 mg/kg of rocuronium. Then the insertion of laryngeal mask will be performed and the pulmonary ventilation will be maintained with 6 ml/kg of ideal-weight and maximum peak pressure 25 cmH2O. The anesthesia will be maintained with sevoflurane in 50% oxygen/air. Sevoflurane concentration was adjusted to ensure an equal depth of anesthesia during surgery as assessed by the bispectral index (BIS; BIS A-1050 Monitor, Aspect Medical Systems, Newton, MA, USA), which will be held between 40-60. Remifentanil will be administered for supplemental intraoperative analgesia and its dose will be adjusted to maintain blood pressure and heart rate within 20% of baseline values. At the end of surgery, residual neuromuscular blockade will be reversed with 2mg/kg of Sugammadex and the laryngeal mask will be removed. Patients will stay on the postanesthetic care unit for at least 1 hour before going to the ward. In the postoperative recovery room, the haemodynamic variables along with postoperative complications such as shivering, hypotension, dizziness, constipation or any other adverse reaction to the drug will be recorded by a blinded observer. After surgery, rescue medication will be intravenous metoclopramide (10 mg) for more than one episodes of nausea or vomiting.

All episodes of nausea and vomiting will be recorded for 48h. Patients will be assessed over 3 time periods: 0-6 h, 6-26 h and 24-48 h after surgery. After discharge, all patients will be contacted by telephone at regular intervals to record the episodes of nausea and vomiting as well as analgesic and antiemetic requirements. The primary outcome measure will include the number of emetic episodes, the incidence of nausea, the need for rescue antiemetics and the analgesic requirement after 48h. Nausea is deﬁned as a subjective unpleasant sensation associated with the urge to vomit without expulsion of gastric content and vomiting is deﬁned as the forceful expulsion of gastric contents trough the mouth.

The secondary outcome will be to evaluate the frequency of polymorphism of the 5-HT3 receptors and their correlation with the antiemetic effect and adverse effects. The patients will have cell samples of the oral cavity, collected through smear. The collected material will be placed in a tube and then will be stored at freezer. Then, it will be analyzed in the laboratory of Clinical Research Unit at the Federal Fluminense University (UFF). After extracting the DNA from the samples using a DNA purification kit, a polymerase chain reaction (PCR) will be performed for analysis of single nucleotide polymorphisms. Biological samples will be discarded at the end of the analysis.

Sample size was calculated with reference to the results of a study comparing weight-adjusted doses of palonosetron for prevention of PONV in laparoscopic gynaecological surgery in no-obese patients. For a P value of 0.05 and a power of 0.8, a minimum of 36 per dose group was required. To allow for possible dropouts, the aim was to recruit 40 patients in each group.

GraphPad Prism 4.0® (GraphPad Softwares Inc., San Diego, CA, USA) will be used for statistical analysis. The Wilcoxon signed rank 2x2 test will be use to ensure that data will be normally distributed. Patient characteristics and intra- and post-operative variables will be analysed by Unpaired Student´s t-test. Proportions will be compared using x^2^ square or Fisher’s exact test. A P value < 0.05 is considered statistically significant.

**Study Administration & Ethical Issues**

The study was approved by the Institutional Ethics Review Committee (Research Ethics Committee (CEP) of INCA), which is formed by professionals from different fields whom review research projects involving human subjects, to ensure the rights, safety and well-being of all volunteers who participate in these studies. Contact the CEP INCA, from Monday to Friday 9:00am to 5:00 pm, by the phones +55 21 3207-4550 and also by e-mail: cep@inca.gov.br.

Some adverse effects may occur with the use of medication, the most frequent being sedation, headache and dizziness. There are also the risks inherent to anesthetic-surgical procedures, such as infection, bleeding, arrhythmias, allergies and pain.
 The information about patient’s health and personal information will be kept private and confidential. The data will only be used anonymous (without identification). Only the authorized researchers will have access to individual´s data, results of examinations and tests as well as the information contained on the medical records. Even if this data is used for scientific publication, patient identification will remain secret. Any and all damages arising from the development of this research and in need of medical care is the responsibility of the institution.

**Study Plan**

The duration of the research will be of twelve (12) months. The data collection phase will be held in six (6) months and the statistical analysis, the writing of a scientific paper and submitted for publication will be done in six (6) months.

### Investigators/Researchers responsables:

Nathalia Gouveia de Araújo Ferreira, M.D., Anesthesiologist. Master student of Department of Anesthesiology of Federal University of Rio de Janeiro, Brazil.

### Nubia Verçosa Figueiredo, M.D., Professor of Department of Anesthesiology of Federal University of Rio de Janeiro, Brazil.

Ismar Lima Cavalcanti, M.D., Professor of Department of Anesthesiology of Federal Fluminense University, Rio de Janeiro, Brazil.

Alexandra Rezende Assad, M.D., Professor of Department of Anesthesiology of Federal Fluminense University, Rio de Janeiro, Brazil

Louis Barrucand, M.D., Professor Faculty of Medicine, Federal University of Rio de Janeiro, Rio de janeiro, Brazil

Estevão Braga, M.D., Professor of Department of Anesthesiology of Federal Fluminense University, Rio de Janeiro, Brazil

Organization's Unique Protocol ID : 55695816.7.0000.5274

### Comparative study between two doses of palonosetron on the prevention of postoperative nausea and vomiting in obese patients undergoing breast surgery
